# Salmonellosis and Meat Purchased at Live-Bird and Animal-Slaughter Markets, United States, 2007–2012

**DOI:** 10.3201/eid2001.131179

**Published:** 2014-01

**Authors:** Maho Imanishi, Tara C. Anderson, Janell Routh, Catherine Brown, Giuseppe Conidi, Lynda Glenn, Vasudha Reddy, HaeNa Waechter, Michelle Malavet, Mawuli Nyaku, Susan Bohm, Sally Bidol, Katherine Arends, Amy Saupe, Jeffrey Higa, Thai-An Nguyen, Jeshua Pringle, Casey Barton Behravesh, Stacey Bosch

**Affiliations:** Centers for Disease Control and Prevention, Atlanta, Georgia, USA (M. Imanishi, T.C. Anderson, J. Routh, M. Nyaku, T.-A. Nguyen, J. Pringle, C. Barton Behravesh, S. Bosch);; Massachusetts Department of Public Health, Boston, Massachusetts, USA (C. Brown, G. Conidi, L. Glenn);; New York City Department of Health and Mental Hygiene, New York, New York, USA (V. Reddy, H. Waechter);; New Jersey Department of Health, Trenton, New Jersey, USA (M. Malavet);; Michigan Department of Community Health, Lansing, Michigan, USA (M. Nyaku, S. Bohm, S. Bidol, K. Arends);; Minnesota Department of Health, St. Paul, Minnesota, USA (A. Saupe);; California Department of Public Health, Gardena, California, USA (J. Higa)

**Keywords:** salmonellosis, Salmonella enterica, bacteria, outbreak, meat, food safety, poultry, livestock, live-bird markets, animal-slaughter markets, foodborne infections, United States

**To the Editor:** *Salmonella* spp. cause ≈1.2 million human illnesses annually in the United States (*1*). Infections are primarily acquired through exposure to contaminated food or infected animals (*1*,*2*). Since 2007, state and local health departments and the Centers for Disease Control and Prevention have investigated multiple salmonellosis outbreaks linked to meat purchased at live-bird markets (LBMs) and live-animal markets (LAMs), where poultry and livestock are sold for onsite slaughter. These markets typically operate in large cities and serve populations of diverse ethnic backgrounds (*3*).

In 2007, an outbreak involving 62 case-patients infected with 1 of 3 *S. enterica* serotype Schwarzengrund strains was investigated in Massachusetts; 61% were children <5 years of age, including 14 (23%) infants <1 year of age, and 96% were Asian (Table). A case-patient was defined as a person infected with *S. enterica* who had a pulsed-field gel electrophoresis *Xba*I restriction enzyme pattern indistinguishable from the outbreak strain. Exposure to poultry purchased at LBMs was reported, and environmental sampling at an implicated LBM identified 6 *S. enterica* serotypes, including 1 outbreak strain.

**Table:** Characteristics of outbreaks of human *Salmonella enterica* serotype Schwarzengrund infections linked to meat purchased at live-bird markets, United States, 2007–2012*

Year	Location	Outbreak strain†	No. cases	No. yes/total no. (%)‡				
				Children <5 y of age§	Infants <1 y of age	Asian race	Hispanic ethnicity	Exposure to meat purchased at live-bird markets
2007	Massachusetts	JM6X01.0240, JM6X01.0225, JM6X01.0118	62	38/62 (61)	14/62 (23)	53/54 (98)	NA	8/10 (80)
2009	New York, NY	JM6X01.0240	50	37/50 (74)	15/50 (30)	7/20 (35)	9/17 (53)	14/16 (88)
2010–2011	Multistate¶	JM6X01.0240	233	105/209 (50)	19/209 (9)	26/72 (36)	29/72 (40)	28/80 (35)
2012	Multistate#	JM6X01.0323	15	8/15 (53)	3/15 (20)	1/12 (8)	5/12 (42)	6/12 (50)

*NA, not available. †Defined by pulsed-field gel electrophoresis *Xba*I restriction enzyme pattern. ‡Denominators are dependent on number of case-patients interviewed and may vary from case counts. §Includes infants <1 y of age. ¶Case distribution includes New York (91, 39%), New Jersey (52, 22%), and Massachusetts (44, 19%). #Case distribution includes Illinois (8, 53%) and Michigan (5, 33%).

Three subsequent investigations of *S. enterica* serotype Schwarzengrund infections were conducted: a 2009 outbreak of 50 cases in New York, New York; a 2010–2011 multistate outbreak of cases predominantly in New York, New Jersey, and Massachusetts; and a 2012 multistate outbreak of cases mostly in Illinois and Michigan. Most case-patients in these outbreaks were of Asian race or Hispanic ethnicity, but 3/5 case-patients in Michigan reported Arab ethnicity; >50% were infants or children <5 years of age.

Among case-patients with available information, exposure to poultry from LBMs was reported by 88% of case-patients in the 2009 New York investigation, 35% in the 2010–2011 multistate investigation, and 50% in the 2012 multistate investigation. In Michigan, the outbreak strain was isolated from chicken purchased at an LBM and collected from households of 2 case-patients.

During 2011–2012, the Centers for Disease Control and Prevention investigated a nationwide increase in *S. enterica* serotype I,4,[5],12:i- infections (pulsed-field gel electrophoresis *Xba*I restriction enzyme pattern JPXX01.1314). Although no single vehicle was implicated, clusters linked to LAMs were identified. In Minnesota, 14 illnesses were linked to meat from 3 neighboring LAMs. Environmental sampling identified the outbreak strain from an animal-holding pen at 1 of the markets. Seven case-patients were infants <1 year of age, and 10 reported Hmong ethnicity. In California, 10 illnesses likely associated with pork, lamb, and beef purchased at 3 LAMs were identified; case-patients reported Ethiopian and Hmong ethnicity. The outbreak strain was isolated from a pork leg collected from the freezer of a case-patient.

LBMs and LAMs appear to be preferred by certain populations for cultural, culinary, or religious reasons. Exposure to meat from these markets is being increasingly recognized as a potential source of salmonellosis. The cause is uncertain, but one factor may be an increased number of markets: in New York, New York, the number of LBMs nearly doubled from 44 to >80 during 1994–2002 (*4*). Most case-patients in these outbreaks had minimal direct contact with poultry or livestock at these markets; many case-patients were infants or young children who had not visited the markets or consumed meat. Therefore, one risk factor appears to be living in a household where the meat purchased from these markets is handled or consumed.

Several factors could make meats from these markets more risky for acquiring salmonellosis. Although LBMs and LAMs must meet sanitation requirements and prevent product adulteration (*5*–*7*), most are exempt from Food Safety and Inspection Service pathogen reduction performance standards (*8*,*9*) and probably do not require suppliers to use pathogen control measures on the farm or employ them during slaughter. Regulatory oversight by state agencies varies. Investigation findings, including environmental sampling, indicate that these markets could be heavily contaminated with *S. enterica*.

Preliminary results of a Massachusetts study found that fresh-killed chickens from LBMs had higher *Salmonella* and *Campylobacter* spp. contamination rates than those for chickens purchased at grocery stores (*10*; T. Stiles, unpub. data). High-risk cultural preferences identified in these outbreaks included consuming raw or undercooked meat and cooking parts (e.g., feet, intestines) that are more likely to harbor *Salmonella* spp. Further processing (e.g., de-feathering, butchering) conducted inside homes could lead to cross-contamination in the household environment. Because of language and cultural barriers, existing food safety messages may not have been effective.

The number and type of LBMs and LAMs, the populations these markets serve, and regulatory authority vary considerably by state, and many case-patients and market owners have been reluctant to speak with public health authorities. Therefore, illness prevention requires a local, targeted approach. To strengthen regulations, some states have created guidelines and begun regular inspection of these markets. Educational outreach has included distribution of posters, flyers, and magnets with safe food handling messages in multiple languages; collaboration with community groups; and education of market owners and workers. Given the various communities who use LBMs and LAMs, multifaceted interventions, including collaboration between human and animal health agencies, are needed to reduce disease risk among market patrons and their families.
